# Engineering a human P2X2 receptor with altered ligand selectivity in yeast

**DOI:** 10.1016/j.jbc.2024.107248

**Published:** 2024-03-29

**Authors:** Elizabeth C. Gardner, Caitlin Tramont, Petra Bachanová, Chad Wang, Hannah Do, Daniel R. Boutz, Shaunak Kar, Boris V. Zemelman, Jimmy D. Gollihar, Andrew D. Ellington

**Affiliations:** 1 Department of Molecular Biosciences, Center for Systems and Synthetic Biology, The University of Texas at Austin, Austin, Texas, USA; 2 Antibody Discovery and Accelerated Protein Therapeutics, Department of Pathology & Genomic Medicine, Houston Methodist Research Institute, Houston, Texas, USA; 3 Department of Neuroscience, Center for Learning and Memory, The University of Texas at Austin, Austin, Texas, USA

**Keywords:** ligand-gated ion channel, yeast humanization, genetic engineering, P2X2, directed evolution, synthetic biology

## Abstract

P2X receptors are a family of ligand gated ion channels found in a range of eukaryotic species including humans but are not naturally present in the yeast *Saccharomyces cerevisiae*. We demonstrate the first recombinant expression and functional gating of the P2X2 receptor in baker’s yeast. We leverage the yeast host for facile genetic screens of mutant P2X2 by performing site saturation mutagenesis at residues of interest, including SNPs implicated in deafness and at residues involved in native binding. Deep mutational analysis and rounds of genetic engineering yield mutant P2X2 F303Y A304W, which has altered ligand selectivity toward the ATP analog AMP-PNP. The F303Y A304W variant shows over 100-fold increased intracellular calcium amplitudes with AMP-PNP compared to the WT receptor and has a much lower desensitization rate. Since AMP-PNP does not naturally activate P2X receptors, the F303Y A304W P2X2 may be a starting point for downstream applications in chemogenetic cellular control. Interestingly, the A304W mutation selectively destabilizes the desensitized state, which may provide a mechanistic basis for receptor opening with suboptimal agonists. The yeast system represents an inexpensive, scalable platform for ion channel characterization and engineering by circumventing the more expensive and time-consuming methodologies involving mammalian hosts.

Ion channels mediate the flow of ions across biological membranes to control resting membrane potential and action potentials. In addition to their native role, ion channels have also been harnessed as genetic tools for the manipulation and analysis of cell function. For example, ion channels have been used for the chemogenetic and optogenetic control of cells (1, 2, 3, 4). However, ion channel engineering poses significant challenges for several reasons. First, ion channels are typically expressed in mammalian cells, which can be slow and expensive to maintain and scale. Second, the measurement of ion channel activity is difficult to parallelize, especially since it may be necessary to measure ion channel activity in real time (5, 6). Finally, the rational design of ion channels is often nontrivial since the relationship between ligand binding and pore opening is often mediated through complex allosteric interactions (7). Therefore, throughput is often a bottleneck to ion channel screening campaigns (8).

An alternative strategy to engineer ion channels is to use the model organism *Saccharomyces cerevisiae* as a host since this microbe is cheaper and easier to cultivate. This species is a model organism with a well-characterized genome, inexpensive propagation, and established tools for genetic engineering and phenotyping (9, 10). Despite the considerable evolutionary distance between humans and yeast, a growing body of evidence has shown that a large fraction of human proteins can be functionally transplanted into yeast (11, 12).

While yeast has been previously used for screening of some mammalian ion channels, these methods have traditionally relied on cell survival phenotypes. For example, active potassium channels have been selected through genetic complementation (13). Another example is constitutively active TRPV1-mediated negative selection in yeast (14). However, these growth-based selections fail to capture key dynamical information such as the rate of channel opening and closing, and the relative magnitude of ionic currents. Instead, live cell assays that use fluorescent ion or voltage indicators can provide such information that may have important implications for downstream cellular control.

One particularly attractive candidate ligand-gated ion channel for yeast expression is the P2X family of purinergic ion channels, which consists of P2X subtypes 1 to 7 (15) These receptors are involved in nociception (16), immunomodulation (17, 18), taste (19, 20), and hearing (21). P2X has been linked to deafness (5), cardiac dysfunction (22), and cancer (23). For the purposes of engineering ion channels in yeast, these receptors are promising candidates for recombinant expression because (a) most of the subtypes form a homotrimer, which simplifies channel expression and assembly and (b) they are cation-nonselective and can thus be assayed *via* common calcium indicators.

Here, we show an inexpensive ion flux assay of a human P2X2 receptor expressed in the yeast *S. cerevisiae*. We demonstrate that this receptor not only shows functional gating in yeast, but that kinetics and rank order of agonist potencies are like those measured in native hosts. We also demonstrate this platform’s promise as a testbed for genetic screening by testing P2X2 mutations associated with hearing loss. Finally, we engineer receptor mutants and create a P2X2 variant, P2X2 F303Y A304W, which is activated by the ATP analog AMP-PNP. Our methodology and our mutant receptor may be a starting point for future engineering campaigns aiming to create unnatural properties. Ion channels engineered with alternative ligand selectivity may be useful for orthogonal cellular control.

## Results

### P2X2 receptors expressed in yeast are functional

**Figure 1:**
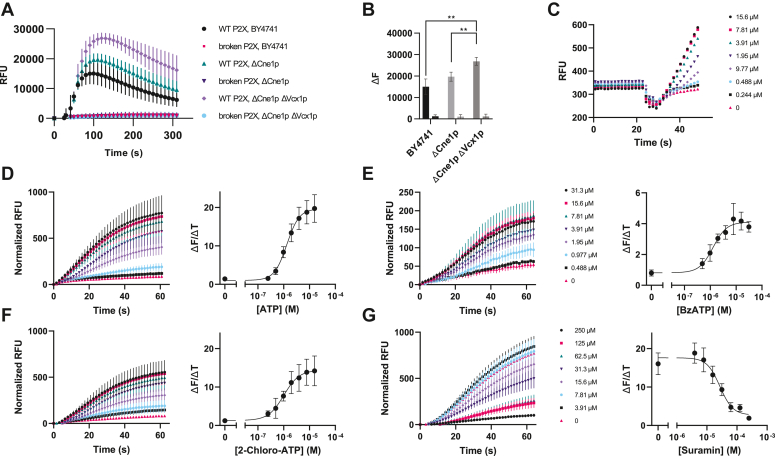
**P2X expression and function in *S. cerevisiae*.***A*, WT-P2X2 yeast *versus* the broken K81C K83C P2X2 mutant after injection of saturating (500 μM) ATP. Genetic knockouts of the calnexin homolog (ΔCne1p) and vacuolar calcium transporter (Δ Vcx1p) (n = 3) were compared to the WT yeast strain (n = 2). *B*, max change in fluorescence from the knockout strains. ΔCne1p and ΔCne1p ΔVcx1p gave significantly improved signal amplitudes compared to WT and was used for all subsequent assays. Statistical analysis was performed using one-way ANOVA with Dunnett’s multiple comparisons test where ∗∗ is *p* ≤ 0.01. *C*, dose response of P2X2 in a shortened GCaMP kinetic assay shows a dose-dependent relationship on the rate of signal onset. Yeast were injected with ATP at 21 s and onset rates from the last six points (40–48 s) were used for calculating the dose response in (D). *E* and *F*, kinetic and dose–response measurements of P2X2 with the GCaMP reporter with ATP (n = 4), partial agonist BzATP (n = 3), and agonist 2-chloro-ATP (n = 4). Figures (*D* and *F*) share the figure legend with (*C*). *G*, inhibitor dose–response with suramin blocks ATP-stimulated gating of P2X2 (n = 4). Suramin was pre-equilibrated with P2X2-yeast for 10 min and 5 μM ATP was injected.

Although P2X receptors are expressed in many eukaryotic organisms including some fungi (24), they are not present in *S. cerevisiae*. For recombinant expression to yield functional P2X channels, receptor proteins must be correctly folded, assembled into trimers, and efficiently trafficked to the plasma membrane. To assess whether P2X ion channels could functionally gate in *S. cerevisiae* the canonical isoforms of P2X1-7 were co-expressed with aequorin, a calcium-activated photoprotein from the jellyfish *Aequorea victoria*. Initial receptor screening was performed in aequorin strains, and P2X2 showed a dose-dependent response several orders of magnitude higher than the other receptors in saturating ATP levels (500 μM) (Fig. S1, *A* and *B*). After the initial receptor screening campaign, we then switched from the aequorin reporter to a fluorescent GCaMP reporter (25) which does not require luciferin incubation steps and is therefore more compatible with high-throughput screening in plate-based assays. In the GCaMP strain, P2X2 showed a 12-fold higher maximum fluorescence compared to a negative control receptor (26) (Fig. 1*A*). The negative control receptor is mutated at two lysines known to directly interact with the ligand, and this receptor variant indeed showed negligible activity in our assay. In addition, we observed no signal when cells are incubated with calcium-free buffer (Fig. S1*A*) and that there is a dose-dependent relationship between signal amplitude and extracellular calcium levels (Fig. S1*B*). These findings suggest that the cytosolic calcium spikes originated with outer membrane-bound channels rather than organellar calcium. These results strongly indicate that the mammalian P2X2 receptor can functionally gate when expressed in *S. cerevisiae*.

As further validation of P2X2 function in yeast, we sought to investigate whether P2X2 could trigger the calcineurin pathway, which is a native pathway controlled by calcium. After intracellular calcium levels rise, calcineurin regulates the nuclear localization of transcription factor Crz1p (27). To test if unnatural ion channels could activate this pathway, a GFP-tagged Crz1p was co-expressed with P2X2 in yeast. Five minutes after ATP injection, nuclear localization of Crz1p was observed in the WT-P2X2 strain (Fig. S2). The degree of nuclear localization was double that of a negative control mutant P2X receptor.

### Strain optimization enhances signal-to-noise ratios and signal duration

To maximize the sensitivity of the GCaMP assay, yeast genome modifications were performed. Since overexpression of heterologous transmembrane proteins *via* multicopy plasmids can trigger the unfolded protein response (UPR) (28), P2X2 was expressed from a single copy in the host genome. In addition, the calnexin homolog chaperone (Cne1p) was deleted, which is a strategy previously shown to increase the expression of other transmembrane receptors (28, 29). Nonetheless, these modifications led to an insignificant increase in signal. To increase the dynamic range of our assay, we sought to limit the sequestration of calcium into vacuoles by deleting the Vcx1p antiporter (30, 31). Deletion of Vcx1p led to a significant increase in calcium signaling following P2X2 opening (Fig. 1, *A* and *B*). Collectively, these strain modifications resulted in an approximately 2-fold increase in signal and increased the duration of the signal by over five-fold.

### Slope of signal onset is a proxy for channel function

To expand the assay throughput to accommodate several 96-well plates at a time, we required the acquisition of short-duration signals. Since GCaMP signals are typically measured in terms of ΔF/F_0_, where ΔF is F_max_-F_min_, and since the signal takes several minutes to reach F_max_, we instead attempted to correlate the *rate* of signal onset (rather than its amplitude) with channel activity. A time course assay was carried out where the signal was measured for less than 30 s after injection (see Experimental procedures), and during the last several seconds of signal onset, the rate of signal accumulation was linear (Fig. 1*C*). We repeated our dose response in the rapid assay format by measuring signaling onset rate (ΔF/ΔT) and recorded an EC_50_ of 1.3 μM (pEC_50_ 5.88 ± 0.056) (Fig. 1*D*), a value which closely resembles previously published values from human glia (6.0 μM) (32). Our rapid assay format allowed us to measure one 96-well plate in 6 min (see Experimental procedures).

Having optimized our yeast strains and assay throughput, we sought to further validate the function of human P2X2 in the yeast host. We performed kinetic assays with P2X2 agonists 2-Chloro ATP (2-Chloro-Adenosinetriphosphate) and BzATP (2′(3′)-O-(4-Benzoylbenzoyl)adenosine-5′-triphosphate) showed appropriate agonist behavior, with EC_50_ values of 1.00 μM (pEC_50_ 6.00 ± 0.11 M) for 2-Chloro ATP (compared to 2.5 μM in mammalian cells) (33) and 1.28 μM for BzATP (pEC_50_ 5.89 ± 0.096 M) (compared to 5–20 μM in mammalian cells) (33, 34, 35) (Fig. 1, *E* and *F*). Partial agonist BzATP exhibited peak amplitudes less than 50% of the canonical ATP ligand in accordance with previous findings (35). Conversely, ATP-mediated P2X2 gating was inhibited by the antagonist suramin with IC_50_ 27 μM (IC_50_ 4.57 ± 0.66 M) (Fig. 1*G*), similar to reported IC_50_ values of 10 μM (36).

### Yeast as a chassis for screening P2X2 mutations of clinical importance

The success of the yeast-based assay in recapitulating wild-type P2X2 function suggested that this model could be used to study proteins involved in human channelopathies. In humans, P2X2 is expressed in cochlear cells and has been associated with autosomal-dominant, non-syndromic deafness (DFNA). Two mutations have been mapped to the P2X2 locus: one identified in Chinese families corresponding to a missense mutation, V60L (37), and one found in Italian families corresponding to a G353R mutation (38). Previous studies suggested that gating is disrupted in these variants (37), and we accordingly performed site-saturation mutagenesis at these sites to determine whether the observed human phenotypes would be recapitulated in yeast.

**Figure 2:**
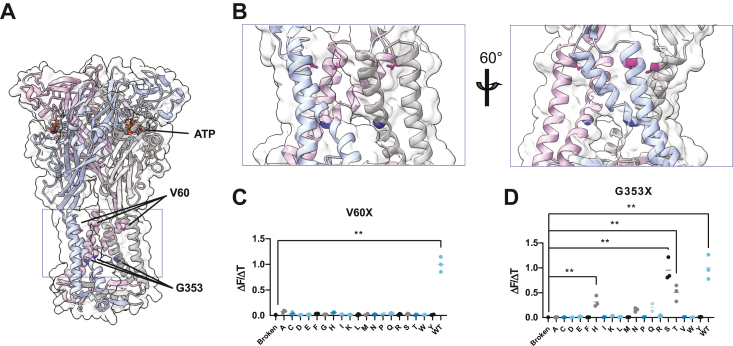
**P2X2 SNP testing.***A*, homology model of P2X2 with the clinically implicated SNPs highlighted. V60 is highlighted in *magenta* and G353 is highlighted in *dark blue*. *Lines* indicate the positions of the mutations on each of the three monomers. *B*, zoomed view of the transmembrane region from the *purple box* in (*A*). V60 is *magenta* and G353 is *dark blue*. The structure is rotated 60° on the right image. *C*, screening data from site-saturation of the V60 residue in saturating ATP concentration (500 μM f.c.) using the fast kinetic assay. Signal onset values (ΔF/ΔT) are normalized to values from the WT receptor. Screening was performed with the GCaMP-P2A-mScarletI configuration of the reporter as a quality control, and replicates that failed to reach a predetermined mScarletI fluorescence standard were discarded from further analysis (See Experimental procedures). Each of 2 to 3 replicates is represented as a single point. *D*, screening at the G353 residue shows three significantly active variants in addition to WT. Statistics were performed *via* one-way ANOVA with Dunnett’s multiple comparisons with to the “broken” K81C K83C negative control. ∗ represents a *p* value ≤0.05, ∗∗ is *p* ≤ 0.01, and ∗∗∗ is *p* ≤ 0.001.z.

The V60 residue is located in the first transmembrane domain and more broadly is part of a pore region that significantly rearranges during channel opening (Fig. 2, *A* and *B*). In the yeast functional screen, all variants at V60, including the V60L mutation, were unresponsive to ATP (Fig. 2*C*), corroborating previous mammalian studies that demonstrated loss-of-function (37, 39), likely due to uncoupling between ATP-binding and pore gating (40). However, when substitutions at G353 were examined, the position proved to be more functionally plastic, with serine, threonine, and histidine residues having significant levels of signaling (95%, 50%, and 32% of WT activity, respectively) (Fig. 2*D*). The clinically observed SNP G353R was not observed to be functional in our assay.

### Deep mutational screening of the ligand-binding domain

**Figure 3:**
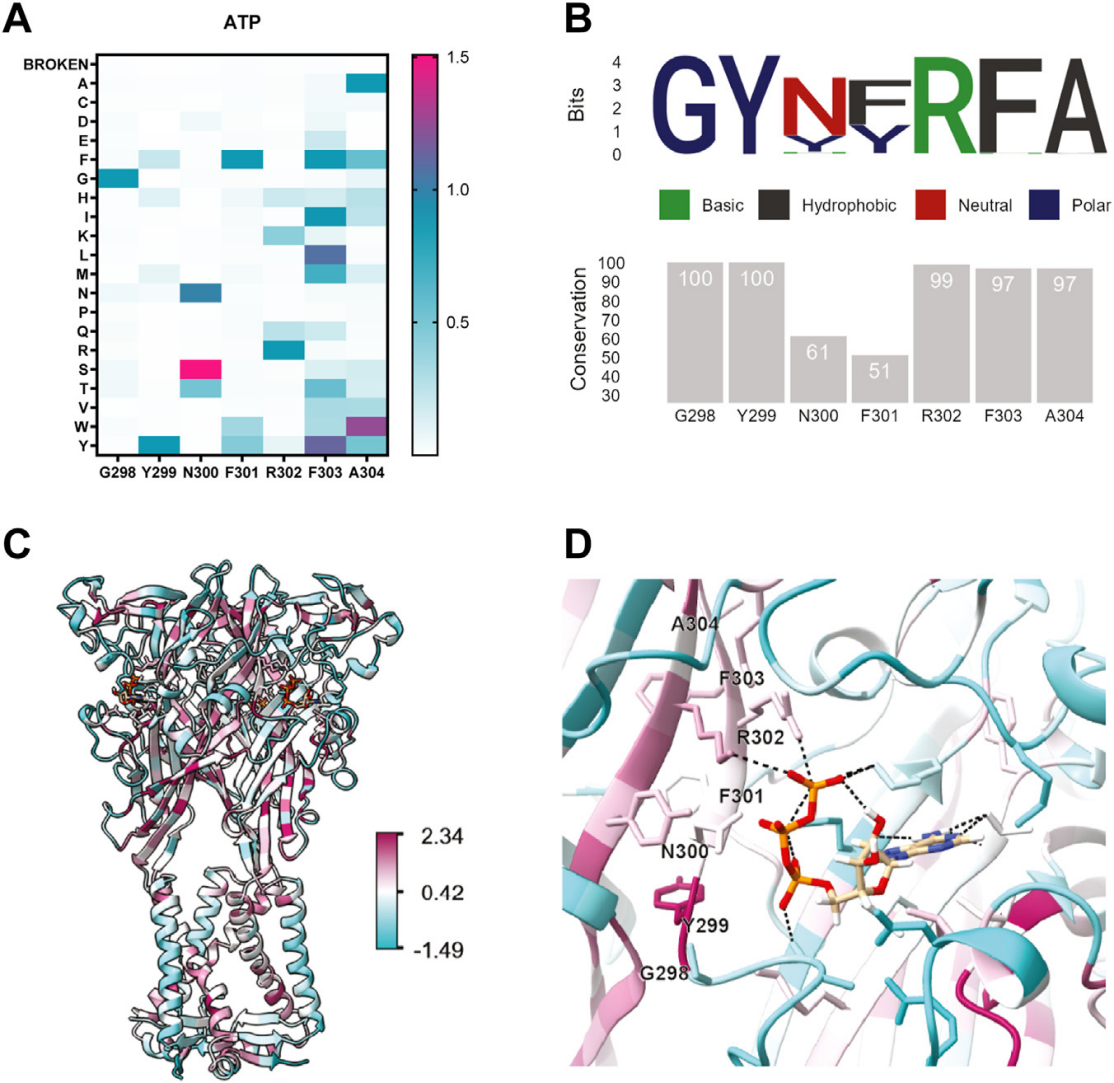
**Structure-function analysis of the ligand binding domain.***A*, heat map summarizing the screening data of ATP with seven site-saturation libraries surrounding the NFR-motif. *B*, sequence-logo plot of the mutagenized residues and percent identity scores as determined from MSA. *C*, homology model of P2X2 colored by sequence conservation as calculated from a multiple sequence alignment of 419 related P2X genes. Conservation is scored on a log scale where maroon is more conserved and *blue* is less conserved. *D*, ATP in the ligand-binding pocket of P2X2. Residues are colored by conservation score. Sites targeted in the library are *highlighted*.

The yeast P2X2 screening platform poses an opportunity to probe the structure-function of the ligand-binding domain in detail. The conserved P2X “NFR” motif at residues 300 to 302 is directly involved in bonding to the ATP triphosphate moiety (41), so we mutagenized residues at or near this motif (298–304), ultimately covering 133 variants (7 sites, 19 residues each). Over these seven positions, G298 and Y299 proved to be the least mutable (Figs. 3*A*, and S5, *A* and *B*), with none of the G298 substitutions exhibiting ligand gating activity compared to the negative control, and only Y299F and Y299H showing function with 21 ± 12% sd and 11 ± 5% sd signal relative to WT, respectively. We compared our results to phylogenetic data based on a multiple sequence alignment (MSA) from a curated set of 419 related P2X homologs (Figs. 3*B*, and S5; Table S1). The phylogenetic analysis showed that residues 298 and 299 were 100% conserved, and were the most conserved residues of the seven examined. A P2X3-based homology model (Fig. 3, *C* and *D*) illustrates that G298 and Y299 likely do not directly bond to ATP despite their high degree of conservation.

In contrast, residues N300 and F301 were the least conserved in phylogeny at only 61% and 51% conservation, where tyrosine is the second most common residue at both sites (Table S1 and Fig. 3*B*). In our screen, N300T was recorded at 51 ± 10% sd of WT signaling, and N300S showed a higher signal than WT (151 ± 16% sd) (Figs. 3*A* and S5*C*). None of the F301 mutants showed significant signaling (Figs. 3*A* and S5*D*). R302, which is a residue that makes contacts with the oxygen of the γ-phosphate, showed modest function with other polar amino acids glutamine (25 ± 28% sd) and lysine (43 ± 15% sd) (Figs. 3*A* and S5*E*).

**Figure 4:**
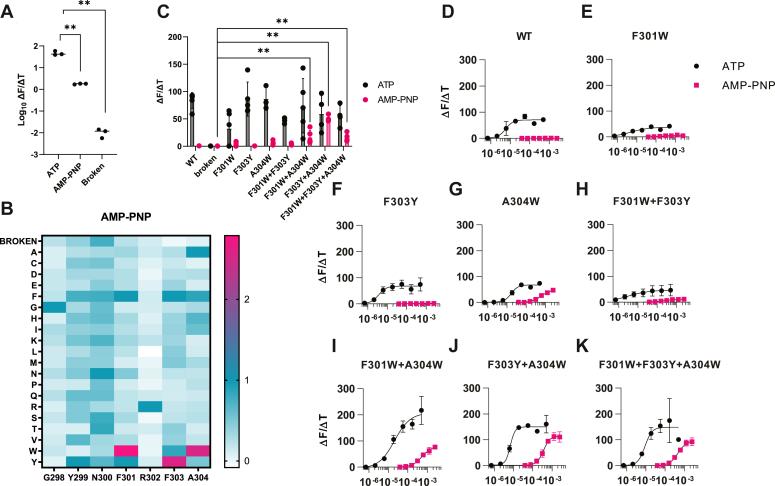
**Unnatural-ligand gating in LBD libraries.***A*, WT P2X2 was tested with 1 mM ATP, 500 μM AMP-PNP, and compared to ATP with the broken receptor (K81C K83C) control with 1 mM ATP in the fast GCaMP-mScarletI assay (n = 3). AMP-PNP shows signaling levels near zero, which was not significantly different from a broken receptor control by a one-way ANOVA. *B*, heat map summarizing AMP-PNP signaling (ΔF/ΔT, fast GCaMP-mScarletI assay) in the site-saturation libraries (n = 2–3). All values are normalized to WT P2X2 with saturating amounts of AMP-PNP (500 μM) and therefore represent low signal (ΔF/ΔT) compared to the ATP ligand. *C*, F301W, F303Y, and A304W were combinatorially combined and tested against the WT, K81C K83C broken P2X2, and the single mutant clones with both ATP (1 mM, *black*) and AMP-PNP (500 μM, *pink*). Double mutants F301W + A304W and F303Y + A304W, and triple mutant F301W + F303Y + A304W had significantly increased signaling capabilities than the negative control. Values here represent signal onset and are not normalized to a positive control (n = 2–5). *D*–*K*, dose response of each P2X variant with both ATP (*black*) and AMP-PNP (*pink*) in the fast GCaMP-mScarletI assay (n = 3). EC_50_ values and summary statistics are summarized in Table S2. Statistics were performed *via* one-way ANOVA with Dunnett’s multiple comparisons with to the “broken” K81C K83C negative control. ∗ represents a *p* value ≤0.05, ∗∗ is *p* ≤ 0.01, and ∗∗∗ is *p* ≤ 0.001.

Residues F303 and A304W do not directly bind ATP but extend the beta-sheet formed by the NFR motif. While these residues are 97% conserved in our phylogenetic analysis, our screening results indicated that these two residues are the most functionally plastic of the group. F303 had four significantly active mutations including F303I (90 ± 42% sd), F303L (108 ± 89% sd), F303M (71 ± 14% sd), and F303Y (113 ± 45% sd) (Figs. 3*A* and S5*F*). A304W had six mutants with significant signals, of which most were bulky hydrophobic residues: A304F (57 ± 2.6% sd), A304H (27 ± 18% sd), A304I (24 ± 17% sd), A304V (32 ± 2.1% sd), A304W (125 ± 47% sd), and A304Y (52 ± 10% sd) (Figs. 4*A* and S5*G*).

### Engineering alternative ligand selectivity

Given that the yeast-based screen seemed to recapitulate important structural and functional features of human ion channel function, we hypothesized that it might be possible to engineer new P2X2 functionalities in yeast, including new ligand specificity. The non-hydrolyzable ATP analog AMP-PNP was chosen as a potential orthogonal signaling ligand, since the wildtype P2X2 receptor shows a nearly undetectable signal with the AMP-PNP molecule (Fig. 4*A*), and because our previously designed NFR motif library should interact with the triphosphate moiety. We screened our 133-member library in the presence of 500 μM AMP-PNP (Fig. S7). While the AMP-PNP signal was uniformly low, three substitutions (F301W, F303Y, and A304W) increased signaling 2.5 to 2.8 times relative to the negative control receptor (Figs. 4*B*, and S7, *D*, *F*, and *G*). To gain a more complete view of the properties of these mutants, dose–response curves were generated with ATP and AMP-PNP (Fig. 4, *D*–*G*, and Table S2). The variants showed modest decreases in signaling with ATP (53–96% of saturated wild-type signal amplitude), and comparable ATP sensitivities, with most EC_50_ values falling within the low micromolar range (F301W EC_50_: 5.04 × 10^−6^ ± 2.12 × 10^−6^ M, F303Y EC_50_: 3.60 × 10^−6^ ± 9.63 × 10^−7^ M, A304W EC_50_: 1.66 × 10^−6^ ± 1.94 × 10^−6^ M, n = 3). When compared to the wild-type, the three variants (F301W, F303Y, and A304W) initially identified in the screen reproduced improved activities with AMP-PNP (F301W: 5.28-fold, F303Y: 1.89-fold), and A304W showed particularly good activation by AMP-PNP at high ligand concentrations (55.87-fold) (Fig. 4, *D*–*G*, and Table S2).

Building on these results, the individual substitutions F301W, F303Y, and A304W were combined as double and triple substitutions, and the four additional variants (F301W + A304W, F303Y + A304W, F301W + F303Y, and F301W + F303Y + A304W were screened with AMP-PNP (Fig. 4, *C*, *H*, *I*, *J*, and *K*, and Table S2). Three of these variants showed significantly improved signaling capabilities: F301W + A304W, F303Y + A304W, and F301W + F303Y + A304W. In particular, F301W + A304W and the triple substitutions showed 24-fold improvement with AMP-PNP compared to the WT channel (rate of signal onset ΔF/ΔT at 500 μM), while the F303Y + A304W variant showed a remarkable 73-fold increased signal. The combined mutations were synergistic, with F303Y + A304W having a predicted additive effect of ca. Ten-fold, relative to the 73-fold seen. Despite high signal amplitudes, the sensitivity of these variants to AMP-PNP lower than the ATP counterpart (F301W + A304W EC_50_: 5.46 × 10^−6^ ± 6.66 × 10^−5^ M, F303Y + A304W EC_50_: 3.53 × 10^−6^ ± 3.05 × 10^−5^ M, and F301W + F303Y + A304W EC_50_: 4.68 × 10^−6^ ± 4.62 × 10^−5^ M).

Notably, all the combinatorial variants except F301W + F303Y showed maximum signaling values with ATP that were 2 to 3 times higher than the wild-type (Fig. 4, *H*–*K*, and Table S2). This finding is in accord with our previous observation that the individual substitutions F303Y and A304W both showed enhanced amplitudes with ATP.

### Identification of a non-desensitizing mutant channel

**Figure 5:**
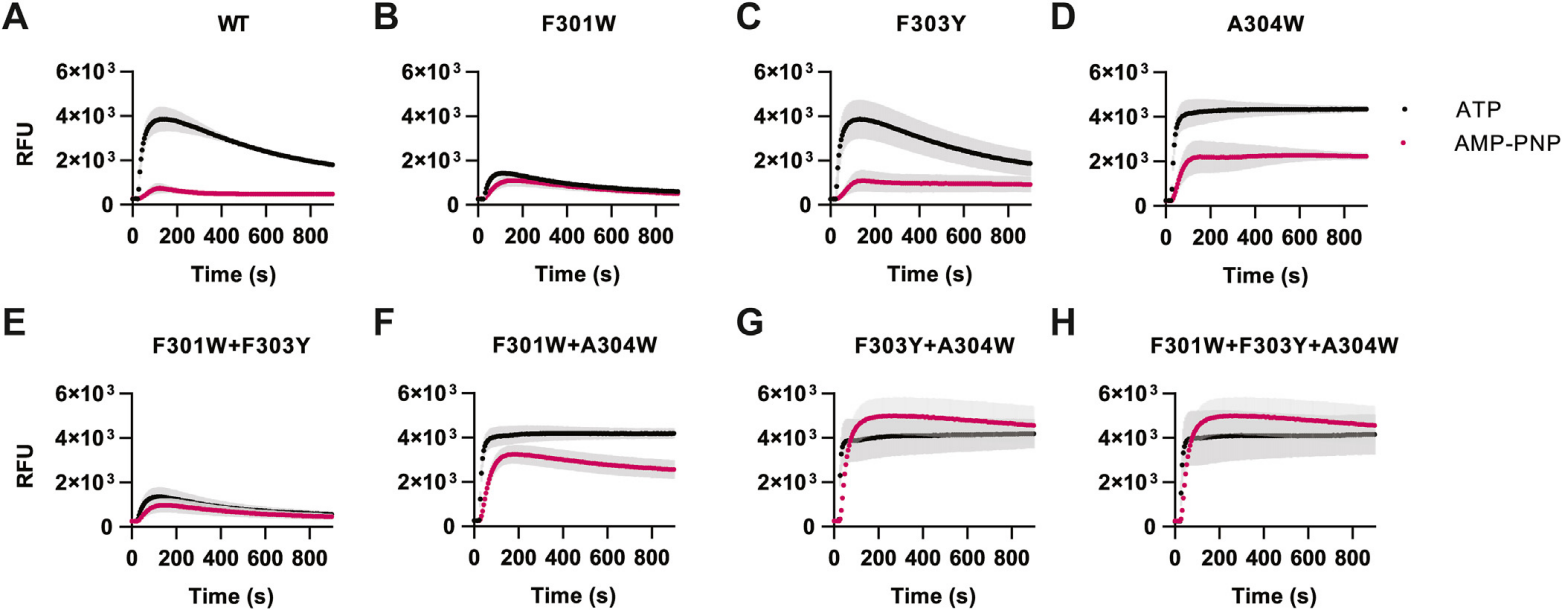
**Kinetic analysis of P2X variants.***A*–*H*, P2X2 variants in GCaMP-mScarletI yeast were injected with 1 mM ATP (*black*) or 5 mM AMP-PNP (*pink*) and fluorescence was monitored over a 15-min time course of bath incubation. Error bars represent standard deviation of the mean (n = 3).

To more fully characterize the selected and engineered P2X2 variants, kinetic assays were performed over a longer period (15 min) that provided a more complete view of receptor gating (Fig. 5, *A*–*H*). The wild-type receptors, F301W and F303Y, showed a steady signal decay over the 15-min timespan, likely caused by receptor desensitization and channel closing.

In contrast, A304W and all combinations (Fig. 5, *D*, *F*, *G*, and *H*) that contained it showed a phenotype in which the channel did not reset, and the signal remained relatively constant throughout the time course. In the presence of ATP, the A304W variant appeared to be unable to close, showing no sign of decay. With the less active AMP-PNP ligand, a small amount of signal decay was apparent. The A304W mutation therefore appears to either favor the open conformation of the receptor and/or to have slow rates of pore closure.

We then performed a structural analysis of the A304W mutation to rationalize our observations. We generated P2X2 homology models with the A304W mutation in the open, closed (apo), and desensitized state. In the energy-minimized homology model, the A304W appears to terminate the beta-sheet and flip the side chain orientation relative to the WT structure (Fig. S8, *A* and *B*). To determine whether this might affect the conformational energetics of the receptor, we performed molecular modeling for each conformation and assessed the potential energy of each substitution in the trimeric receptor. We calculated that while the A304W mutation stabilizes the open state by −4.0 kcal/mol and the closed state by −4.5 kcal/mol (Table S3), the desensitized state is destabilized by 1.8 kcal/mol, supporting our experimental observations.

## Discussion

Although *S. cerevisiae* has homologs of human ion channels such as the TRP family and various voltage-gated channels, it does not have any P2X family homologs (42). In this work, we describe the first functional expression of a P2X channel in this host and use a real-time assay to record calcium transients triggered by channel gating cycles. This contrasts with previous works that use growth-based selections (13, 14), which cannot be used to observe real-time gating kinetics. Survival assays typically provide binary answers about channel activity but are less amenable to quantitative comparisons among active receptors and miss key information about gating dynamics. For example, it is not clear that all types of active channels would lead to cell death phenotypes; receptors that quickly desensitize may be protected from selection but would be detectable in the live assay. The real-time assay also overcomes the confounding factor of ligand toxicity since the assay is completed in seconds *versus* hours or days.

This study presents a novel platform for rapidly screening human P2X2 ion channels in real-time, but there are important limitations to consider. Yeast and mammalian hosts are optimal at distinct pH and temperature conditions and may have differences in post-translational modifications or folding. As a result, discrepancies may arise between yeast and human assays, despite our P2X2 assays being largely consistent with previously published data. Additionally, future iterations of the technology could include normalization by accounting for differences in receptor folding/trafficking. Lastly, our current assay does not identify constitutively active receptors, which may be lethal to the yeast. Despite these limitations, our screening results showed broad biochemical consistency, with amino acids possessing similar properties having similar activity.

Building upon our validated screening assay, we used it to perform deep mutagenesis on the receptor to elucidate structure-function relationships. By exploring the full sequence space at each residue, our assay revealed greater functional plasticity of the P2X2 receptor than might have been inferred from phylogeny. For instance, although the NFR motif is perfectly conserved in the human P2X family and broadly conserved across taxa, we found a variety of residues with equal or greater function than WT that were not observed in nature. For example, R302 is highly conserved (99%) in phylogeny, but is readily substituted in yeast; F303 is readily substituted by tyrosine in yeast but is only found once among 419 phylogenetic variants. Overall, while F303 and A304W showed 97% pairwise identity in a multiple sequence alignment, approximately 20 to 30% of non-wild-type residues showed significant signaling compared to the broken receptor. In some instances, amino acid substitutions found in nature did not correspond to activity in our assay: tyrosine and histidine were observed at position 300, but were non-functional in the yeast-based screen. This discrepancy is likely explained by missing sequence context; the MSA showed that tyrosine or histidine at position 300 was nearly always followed by tyrosine at position 301, as opposed to phenylalanine in human P2X2. This underscores the need for expanded mutagenic libraries encompassing multiple mutations in future implementations of the assay.

Beyond applications in basic science, our P2X2 screening platform is perhaps best leveraged for receptor engineering campaigns that require rapid and inexpensive analysis of large sequence spaces. The more tractable yeast host allowed the rapid engineering of ion channel variants with new activities. Variant libraries were screened for activation with AMP-PNP, which otherwise has very low activity with the wild-type receptor. Initial low-activity “hits” were combined to yield channels that were fully activated by AMP-PNP while also retaining various levels of ATP-gating abilities. We found that the residues that contributed to altered ligand selectivity were not predicted to directly bond to the ligand, but were distal (F303Y, A304W) or facing away from the ligand (F301Y). Of these mutations, A304W is distinct because it appears to block receptor desensitization, which is corroborated by our molecular dynamics simulations. We hypothesize that in the A304W receptors, AMP-PNP can inefficiently open the receptor, but that the receptor then achieves high calcium amplitudes because the open receptor cannot efficiently desensitize. Our proposed model of altered ligand selectivity through open-state bias is consistent with similar observations in other mutants; in particular, the North group observed that the F44C mutation in TM1 led to efficient αβmeATP activation (43). Since F44C is seated in the transmembrane domain, it is improbable that it directly affects the ligand binding interaction, but instead is more likely to control the dynamics of pore opening. In the case of our F301Y A304W mutant, the mechanism behind desensitization blockade is not immediately obvious since these mutations are distant from the intracellular termini canonically associated with desensitization (41, 44). Instead, F301Y and A304W are located just above the ligand binding pocket (see Fig. 3), and while the ectodomain has been linked to desensitization, its role is poorly understood (43, 45, 46, 47). Therefore, our work highlights an unconventional mechanism by which residues around the ligand binding domain can control not only ligand selectivity but also conformational dynamics.

Our rapid, inexpensive, and accessible screening methodology presents a valuable tool for ion channel analysis and engineering. Using this approach, we were able to perform deep screening of the sequence space at and around the ligand-binding domain of the human P2X2 receptor and discovered non-obvious mutations that affect ligand binding selectivity and receptor desensitization. Our engineered F303Y A304W mutant receptor can be fully activated by AMP-PNP, making it a promising candidate for future engineering campaigns aimed at creating orthogonal chemogenetic receptors. We anticipate that our yeast-based approach can be expanded to other ion channels for expression, function, and engineering. Overall, our methodology offers a rapid, inexpensive, and scalable solution for ion channel analysis that may democratize ion channel engineering.

## Experimental procedures

### Molecular biology

Cloning was performed from the MoClo yeast toolkit (48) library and workflow. Parts not included in this library are described separately (Table S4)). Plasmid assemblies were assembled from yeast toolkit parts and novel parts as described (Table S5).

Site-saturation mutagenesis was performed with overlap extension PCR using NNS primers (IDT). Amplicons were cloned into a custom GFP dropout vector (EG.B.445) by golden gate assembly using BsaI-v2 (NEB). 192 colonies were sequenced, and clones were chosen for each amino acid mutation at a particular residue.

Golden gate assembly was used for all cloning. Reactions were performed in a 20 μl reaction as follows: 40 fmol per part, 2 μl 10× T4 ligase buffer (NEB), 1 μl T7 ligase, 1 μl Type IIs restriction enzyme Esp31 or BsaI-v2 (NEB), 1 μl T7 ligase (NEB). Thermal cycling was performed as follows: 37 °C for 2 min, 16 °C for 5 min for 25 cycles, 37 °C for 10 min, 80 °C for 10 min 5 μl each assembly was transformed in DH10B *E. coli* prepared with the Mix and Go *E. coli* transformation kit (Zymo Research).

### Yeast genome editing

Yeast genes were knocked out *via* CRISPR-Cas9 as described elsewhere (48, 49). Briefly, a CRISPR-Cas9 expression construct containing a guide RNA and Cas9 protein was designed (plasmid EG.B155). sgRNA was designed in Benchling (https://benchling.com). sgRNA oligos were annealed and assembled into a sgRNA dropout vector (pYTK050) *via* a golden gate (plasmid EG.B150). Vcx1 sgRNA 5′ oligo: GACTTTGCAATCCAAAGCACACTGCA. Vcx1 3′ oligo: AAACTGCAGTGTGCTTTGGATTGCAA. Repair DNA contained 70 bp 5′ and 3′ homology to the gene locus and introduced an N-terminal in-frame stop codon and barcode for validation purposes. Repair DNA sequence was ordered as a gblock (IDT): AACAACATAGATACAATGGATGCAACTACCCCACTATTAA**CTGTTGCGAACAGTCATCCC**TAAAACTTGCGCTCAATTCCTATGATTTACAGTATATTTTGAAAGCGTCACCCCTGAATTTCCTATTGGTATTTGTTCCT where the underlined region is the stop codon and the bolded area is the barcode. 1 μg of the Cas9-sgRNA plasmid and 5 μg of repair DNA were transformed into yeast using methods described in “Yeast transformations”. Colonies were selected on SD –Leucine and genomic deletion was confirmed by Sanger sequencing (Fig. S9*A*).

### Proteomic validation of deletions

The ΔVcx1p deletion was also validated by liquid chromatography-mass spectrometry (LC-MS/MS) (Fig. S9*B*). Briefly, strains were grown to saturation and pelleted before processing. LC-MS/MS analysis was performed on tryptic peptides separated by reverse phase chromatography on a Dionex Ultimate 3000 RSLCnano UHPLC system (Thermo Scientific) with an Acclaim C18 PepMap RSLC column using a 3 to 42% acetonitrile gradient over 60 min. Peptides were eluted directly into a Thermo Orbitrap Eclipse mass spectrometer by nano-electrospray. Data-dependent acquisition (DDA) was applied, with precursor ion scans (MS1) collected by FTMS at 120,000 resolution and HCD fragmentation scans (MS2) collected in parallel by ITMS with 3-s cycle times. Monoisotopic precursor selection and charge-state screening were enabled, with ions > +1 charge selected. Dynamic exclusion was applied to selected ions ± 10 ppm for 30 s. Proteome database searching and analyses were processed using Proteome Discoverer 2.2. Mass spectra were searched against a protein sequence database containing reversed decoy sequences comprising the *S. cerevisiae* reference proteome (UniProt OX: 559292), a list of common protein contaminants, and plasmid components. See Table S5 for Cas9 and sgRNA plasmid designs. BY4741 Cne1p knockout was acquired from the Stanford *Saccharomyces* Genome Deletion project (Horizon Discovery).

### Multiple sequence alignment

NCBI Protein Reference Sequences (AA) database was interrogated for a BLAST query of Human P2X2 Isoform A protein sequence using Geneious Prime software (Biomatters). 1000 target sequences were identified with an E-value below 0.001. These were filtered for a single protein isoform per organism with the highest similarity grade and lowest E-value. The curated sequence list was realigned in Geneious, and a tree was built using neighbor-joining. Taxonomy annotation was performed using Taxonkit and the tree was visualized in iTOL software (https://itol.embl.de/itol.cgi).

### Homology models

A homology model of P2X2 derived from P2X3 in the ATP-bound, open state (PDB 5svk.1.C) was downloaded from the Swiss-Model (50). The average model confidence was 0.68 ± 0.05. To assess ligand-binding, the homology model was structurally aligned to 5svk which includes the bound ATP ligand. Models were colored by conservation from the multiple sequence alignment described elsewhere. Conservation was calculated using the AL2CO program (51) in ChimeraX software. Homology models were separately created and optimized for each computational simulation. Models were created from P2X3 structures for the open (PDB: 5svk), closed (PDB: 5svj), and desensitized (PDB: 5svl) forms of the receptor. Models were created in Molecular Operating Environment (MOE.09.2015). The structures were inspected for anomalies and protonated/charged with the Protonate3D subroutine (310K, pH 7.4, 0.1 M salt). The protonated structures were then lightly tethered to reduce deviation from the empirically determine structures and minimized using the Amber10:EHT forcefield with R-field treatment of electrostatics to an RMS gradient of 0.1 kcal mol^−1^. Models of P2X2 variants were created by mutating individual amino acids in all three chains of the trimer. The final model of each variant was further refined by placing the protein within a 6 Å water sphere and minimizing the solvent-enclosed structure to an RMS gradient of 0.001 kcal mol^−1^ Å^−1^. Models were evaluated by calculating Phi-Psi angles and superimposed against the reference structures. Agonist structures were created in cleft, and the LigandInteractions subroutine was used to calculate binding energies. Interactions were scored based on stability, affinity, and potential energy of the system.

### Yeast transformations

Yeast were transformed with the Zymo EZ yeast transformation II kit (Zymo Research). For genomic integrations, 2 to 3 μg plasmids were linearized with NotI before transformation. 2 to 3 μg plasmid was digested with 1 μl NotI (NEB) for 1 h at 37 °C and heat inactivated prior to transformation. 5 μl of the digestion reaction was used for 100 μl cells.

### Yeast strains

All strains were based on the BY4741 background (*S. cerevisiae*, S288C derivative) MATɑ his3Δ1 leu2Δ0 met15Δ0 ura3Δ0. See Table S6 for all final strains.

### Functional assays

Aequorin assays were performed as follows. P2X2 aequorin strains were allowed to grow overnight in SD –uracil/-histidine dropout medium. Samples were diluted 1:10 in –uracil/-histidine dropout media with 5% D-galactose and 2% D-raffinose as a carbon source. Samples were grown at 30 °C for 18 h. Cells were washed and the cell wall was “softened” to increase cell wall permeability. Approximately 10^8^ cells were washed in 1 ml water, and then 1 ml softening reagent (1 M sorbitol, 25 mM EDTA, 50 mM DTT). Cells underwent a recovery wash in 1 ml 1 M sorbitol solution. Cells were then incubated with 0.01 μg/μl coelenterazine in the induction media and gently mixed at 30 °C for 30 min. Cells were centrifuged and the supernatant was discarded. Cells were washed and resuspended to a final volume of 900 μl in 0.1 M MES/Tris buffer pH 6.5 (52) with 5 mM calcium unless otherwise specified. 100 μl cells were loaded into a white microplate and injected at 100 μl/s with 100 μl ligand suspended in the MES/Tris buffer (without added calcium). Luminescence was measured in a Tecan Infinite F500 plate reader.

GCaMP assays were performed as follows. P2X2 GCaMP strains were designed with a bi-cistronic reporter, in which the GCaMP protein was followed by a 2A peptide and a mScarletI reporter (53). The co-translated mScarletI was designed to act as a control for plasmid loss, GCaMP breakage *via* nonsense mutation, and contamination. Z-scores were calculated to be 0 to 0.5 (54), indicating that replicates during screening were necessary (Fig. S4). P2X2-GCaMP strains were cultured and induced as previously described. Samples were induced for 24 h to allow for the accumulation of the mature GCaMP protein. Cells were washed three times in 0.1 M MES/Tris buffer and resuspended with MES/Tris with supplemental calcium. Cells were loaded onto a black, clear bottom microplate and imaged on a FlexStation3 plate reader. Samples were imaged in “flex” mode. Samples were imaged for 48 s at an interval of 1.6 s. Fluorescence was measured with an excitation at 485 nm, and emission 525 nm. At the 21 s mark, 100 μl ligand solution was transferred to the cells. For strains with mScarlet, fluorescence was read with excitation 570 nm/emission 610 nm. Slopes were calculated from the last 6 points of measurement and were normalized to the positive control samples. Any replicate in which the mScarlet value was measured to be over 2 standard deviations less than the mean for that set was discarded from further analysis.

### Crz1p imaging

Yeast strains EG.y156 WT-P2X2 or EG.y66 broken-P2X2 were transformed with EG.B723 GFP-tagged Crz1p. Yeast were cultured and induced as previously described. Cells were loaded onto a cell imaging microplate (Eppendorf, cat. no. 0030741030) that was pre-treated with Poly-D-lysine (50 μg/ml). Cells (50 μl) were attached to the plate for 30 min at room temperature, and wells were then gently washed three times with media to remove non-adherent cells. Cells were resuspended in 100 μl and imaged on a Cytation 5 Cell Imaging Multi-Mode reader. Cells were imaged before injection with the appropriate substrate, immediately after injection, and 5 or 10 min after injection. 375 μM CaCl_2_ or 200 μM ATP (f.c.) was injected. Nuclear localization events were automatically counted in Ilastik (https://www.ilastik.org/). Percent nuclear localization was counted as nuclear localization events divided by total cells.

### Statistics

For multiple comparisons in the site-saturation screening data, One-way ANOVA with Dunnett’s multiple comparisons test was performed where every sample was compared to the “broken” negative control. Significance was plotted where ∗ represents a *p* value ≤0.05, ∗∗ is *p* ≤0.01, and ∗∗∗ is *p* ≤0.001. Dose–response curves were fit with nonlinear regression software using a four-parameter logistic curve. All statistics were calculated and plotted in Graphpad Prism software.

## Data availability

Any data not included in this manuscript will be made available upon request to the authors.

## Supporting information

This article contains supporting information.

## Conflict of interest

The authors declare that they have no conflicts of interest with the contents of this article.
