# Supplementary material for: Engineering a human P2X2 receptor with altered ligand selectivity in yeast
Source: J Biol Chem. 2024 Mar 29;300(5):107248. doi: 10.1016/j.jbc.2024.107248 (PMC11063903; doi:10.1016/j.jbc.2024.107248)
Supplement: Supporting Figures S1–S9 [file mmc2.pdf]

Supplementary Information

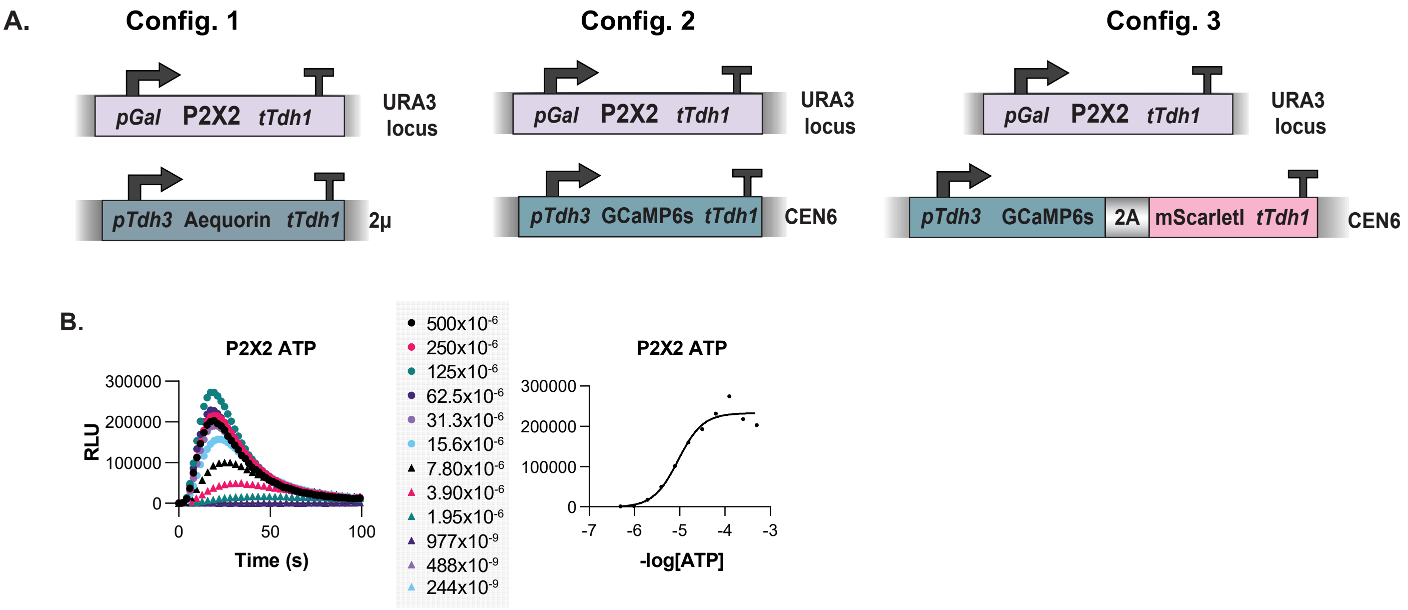

Figure S1: P2X expression and function in *S. cerevisiae* (A) Schematic of P2X2 and reporter genetic constructs are shown. P2X2 was genome integrated under a galactose inducible promoter. Reporter constructs were created in one of three styles referenced throughout this work. Upon ligand binding, functional P2X2 channels open and facilitate an influx of calcium. Intracellular calcium is quantified using either the fluorescent calcium indicator GCaMP or the luminescent indicator aequorin. Yeast were induced with galactose overnight, incubated with luciferin coelenterazine, and finally washed and resuspended with buffer supplemented with 5 mM $CaCl_2$. Receptor activity was measured on a plate reader, where luminescence was measured before and after injection of various ligands. (B) Functional screen (n=1) of P2X2 and the 2μ aequorin reporter with ATP in a 12-point dose response. The Y-axis in the dose response represents the maximum RLU after injection.

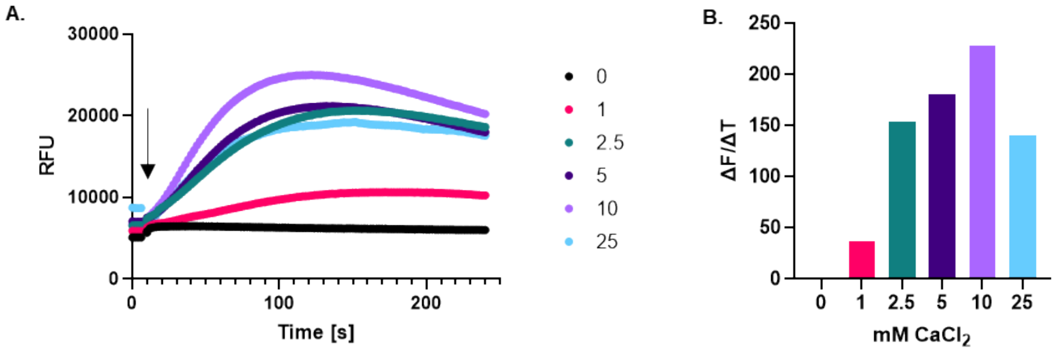

Figure S2. Calcium dependency on P2X2 signaling. A) WT P2X2 with varying levels of extracellular calcium measured with GCaMP fluorescence. 500 μM ATP was injected at the time point indicated by the arrow. B) Slope of signal onset from A).

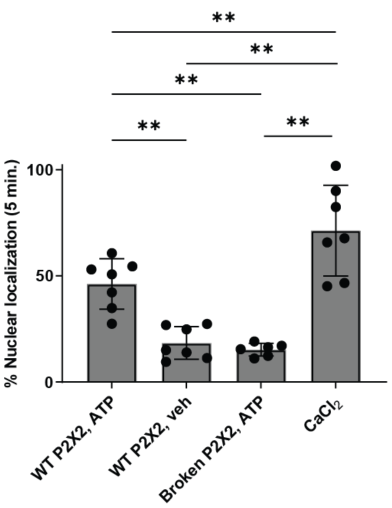

Figure S3: Human P2X2 drives yeast signal transduction. P2X2 with GFP-tagged Crz1p was injected with 100 uM ATP or vehicle. Yeast strain ΔCne1pΔVcx1p was used. “Broken” K81C K83C P2X2 was also injected with ATP. A control strain was injected with extracellular calcium as a positive control for nuclear localization. Percent nuclear localization was measured 5 minutes after injection. One-way ANOVA with Dunnett’s multiple comparisons test was performed where every sample was compared to the “broken” negative control. Significance was plotted where * represents a P value ≤0.05, and ** is P ≤0.01.

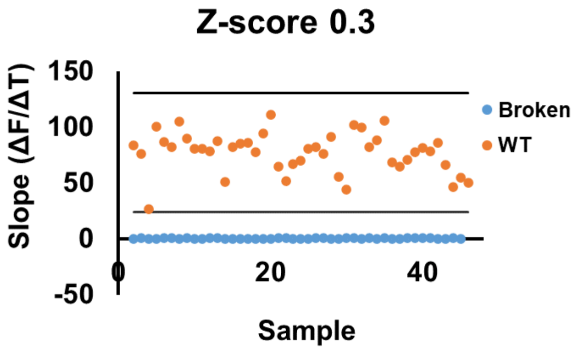

Figure S4. Z-score was calculated from 96 biological replicates containing either the WT P2X2 or the K81C K83C broken gene. Z-score was calculated as defined elsewhere (Zhang, 1999) (45).

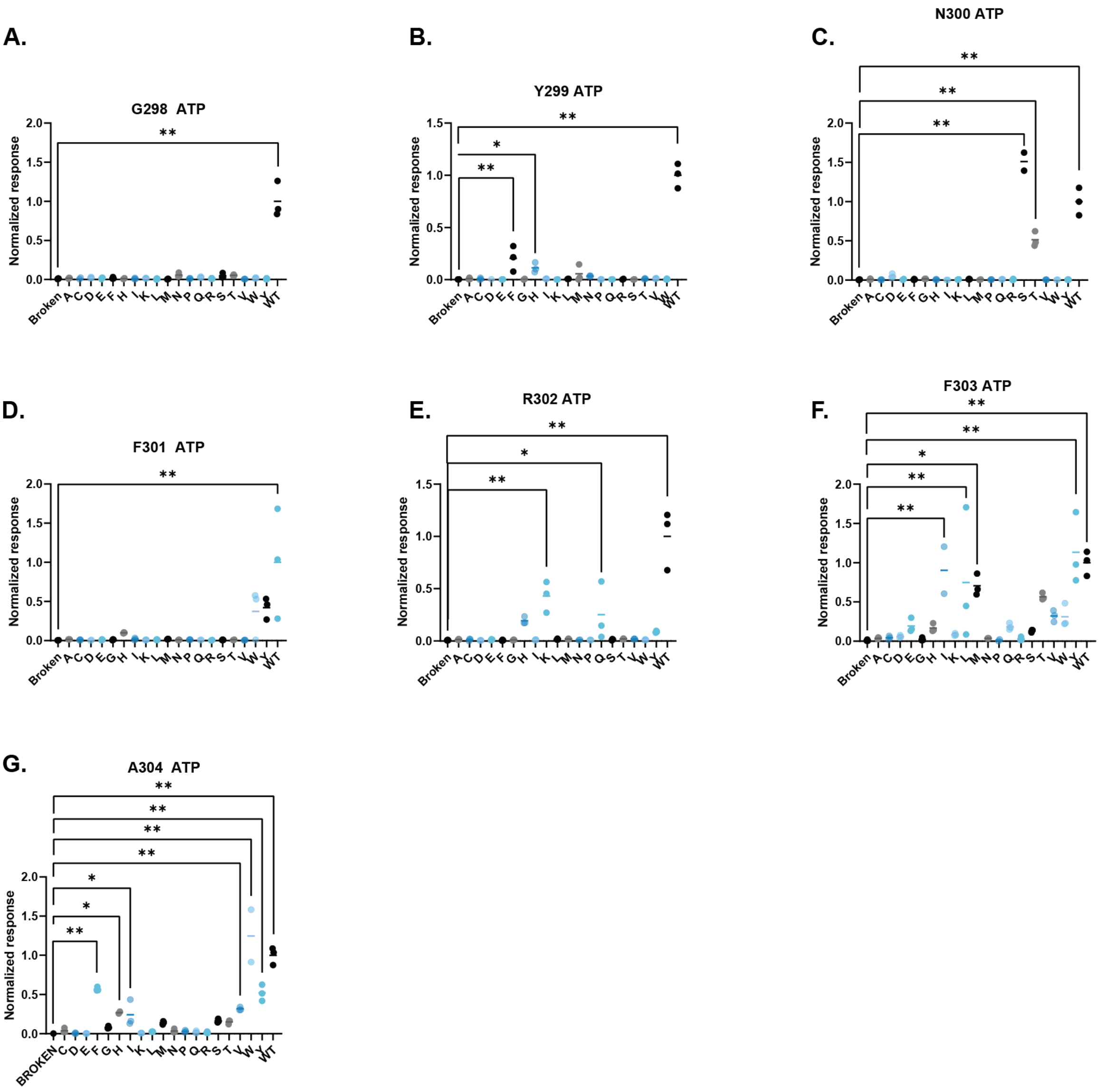

Figure S5. Screening site-saturation libraries of the ligand binding domain with ATP. Seven residues surrounding the NFR domain in P2X2 were site saturated and measured using signal onset rates (ΔF/ΔT). Values were normalized to the WT P2X2 with ATP. Saturating levels of ATP were injected (500 μM). One-way ANOVA with Dunnett's multiple comparisons test was performed where every sample was compared to the "broken" negative control. Significance was plotted where * represents a P value ≤0.05, and ** is P ≤0.01.

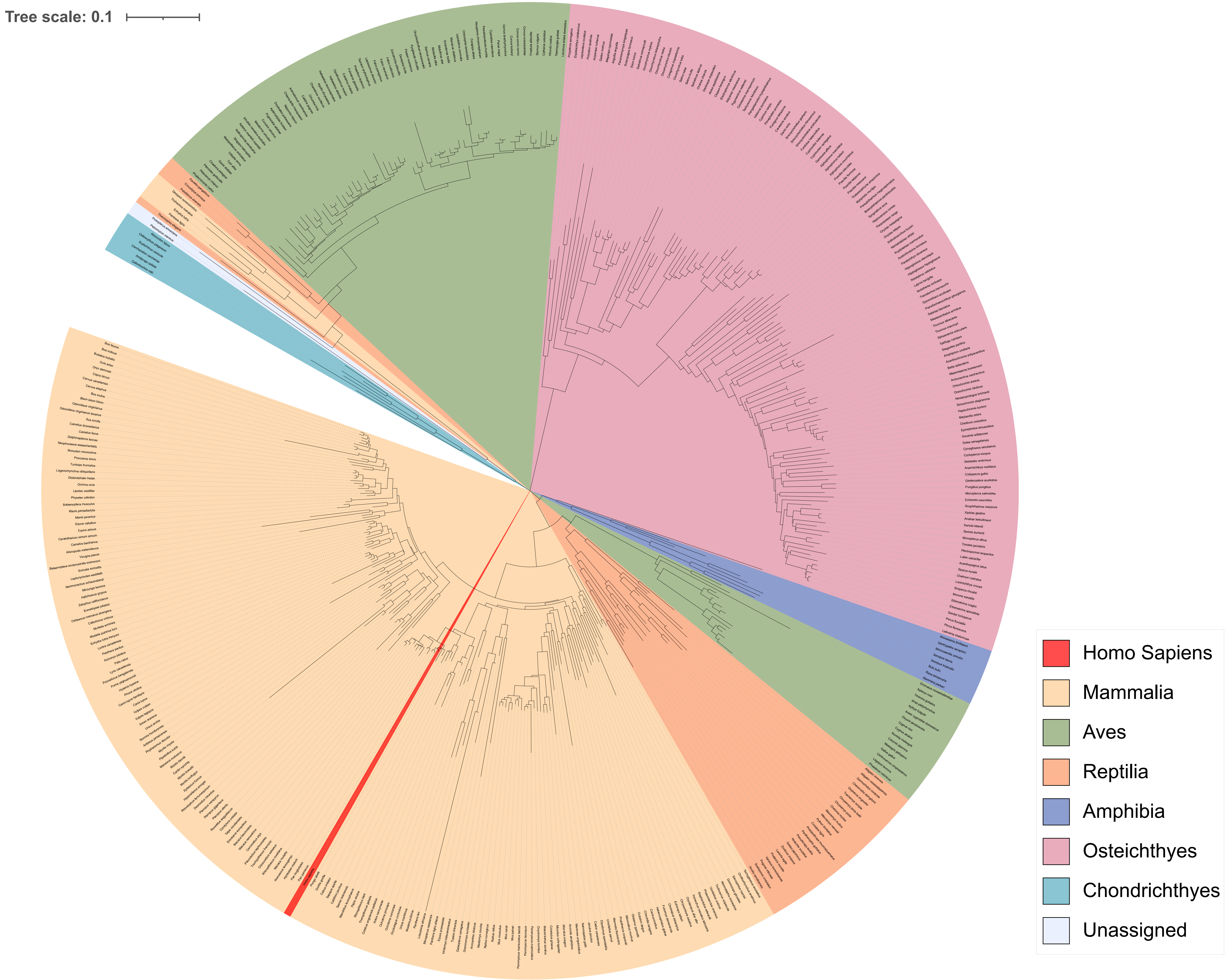
Tree scale: 0.1
Homo Sapiens
Mammalia
Aves
Reptilia
Amphibia
Osteichthyes
Chondrichthyes
Unassigned

Figure S6. Tree of curated P2X2 sequences colored by major taxonomic classes. BLAST query of human P2X2 protein sequence, corrected for isoform bias through removing duplicate organism hits, were clustered by neighbor joining method and visualized as a tree. P2X2 sequences aggregate in agreement with the major taxonomy classes: Mammalia, Aves, Reptilia, Amphibia, Osteichthyes, Chondrichthyes. The Homo sapiens sequence is shown in red.

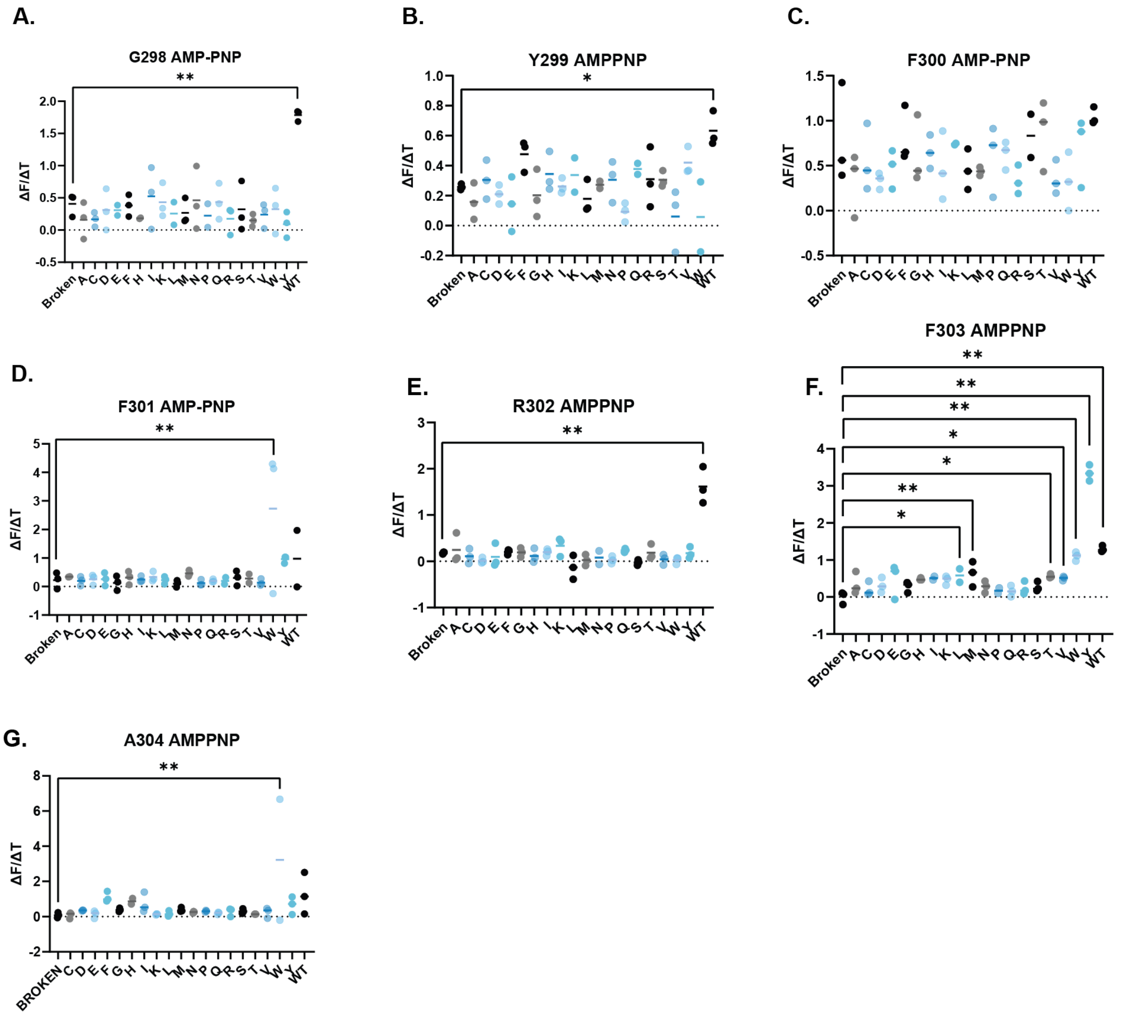

Figure S7. Screening site-saturation libraries of the ligand binding domain with AMP-PNP. Seven residues surrounding the NFR domain in P2X2 were site saturated and measured using signal onset rates (ΔF/ΔT). Values were normalized to the WT P2X2 with AMP-PNP. Saturating levels of AMP-PNP were injected (500 μM). One-way ANOVA with Dunnett's multiple comparisons test was performed where every sample was compared to the "broken" negative control. Significance was plotted where * represents a P value ≤0.05, and ** is P ≤0.01.

A. B.

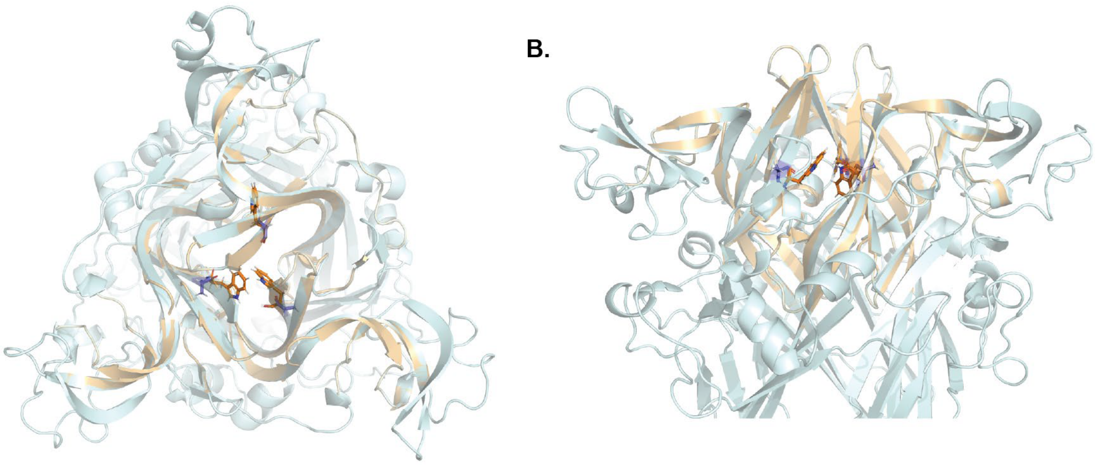

Figure S8. Homology models for WT P2X2 (blue) and A304W P2X2 (orange) were overlaid, and the resides for A304 and W304 were highlighted in opaque color. A) Top view B) Front view.

A.

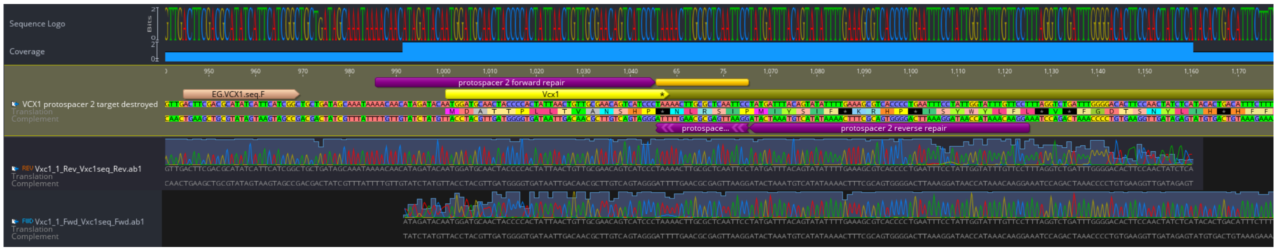

B.

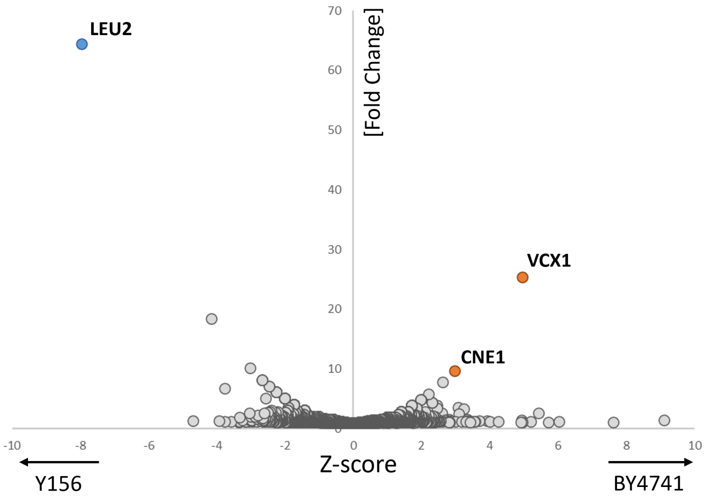

Figure S9. A) CRISPR knockout of ΔVcx1p was validated by Sanger sequencing of the genome. Sequencing was aligned and visualized using Geneious Prime 2023.0.1 B) Differential expression (fold change) between WT yeast strain BY4741 and ΔCne1p Δ Vcx1p knockout strain (Y156). Knockout was validated on a proteomic level via mass spectrometry. Plot shows statistical significance (Z-score) and fold change for the entire set of 4229 proteins observed across both strains. Vcx1p and Cne1p (orange dots) show the greatest fold change in BY4741 compared to the knockout strain, with Vcx1p having a 25.42 fold-change, Z-score 4.94, and Cne1p with a fold-change of 9.78, and Z-score 2.97. The Y156 strain shows increased expression of the Leu2 selection marker.
